# Machine learning for predicting strength properties of waste iron slag concrete

**DOI:** 10.1016/j.heliyon.2025.e42133

**Published:** 2025-01-23

**Authors:** Matiur Rahman Raju, Syed Ishtiaq Ahmad, Md Mehedi Hasan, Noor Md. Sadiqul Hasan, Md Monirul Islam, Md. Abdul Basit, Ishraq Tasnim Hossain, Saif Ahmed Santo, Md Shahrior Alam, Mahfuzur Rahman

**Affiliations:** a Department of Civil Engineering, International University of Business Agriculture and Technology, Dhaka, 1230, Bangladesh; b Department of Civil Engineering, Bangladesh University of Engineering and Technology, Dhaka, Bangladesh; c Department of Computer Science and Engineering, International University of Business Agriculture and Technology, Dhaka, 1230, Bangladesh; d Local Government Engineering Department (LGED), LGED Bhaban, Dhaka, 1207, Bangladesh

**Keywords:** Concrete, Compressive strength, Split tensile strength, Waste iron slag, Machine learning, Cost analysis

## Abstract

This study investigates the utilization of waste iron slag (WIS) as a sustainable alternative in concrete production to reduce environmental impact and preserve natural resources. The experimental investigation of WIS-incorporated concrete focused on compressive and tensile strength with machine learning (ML) models for prediction. Among the tested ML algorithms, Decision Tree (DT) and XGBoost showed the highest accuracy (R^2^ = 0.95135) in predicting concrete strength properties, while models like SVM and Symbolic Regression underperformed. Experimental results indicate that up to 20 % WIS replacement maintains adequate strength, whereas higher proportions reduce structural integrity. A ranking score index and cost analysis confirmed the technical and economic feasibility of using WIS in concrete. Cost analysis demonstrated substantial cost savings with 25 % WIS incorporation, confirming its economic feasibility. Integrating experimental data with ML predictions highlights WIS's potential for sustainable concrete applications, enabling optimized mix designs and reduced reliance on physical testing. Future work should address limitations, including dataset expansion and the exploration of additional durability and mechanical properties to validate WIS's practicality in construction further.

## 1 Introduction

Concrete, a fundamental material in construction, plays a pivotal role in infrastructure development globally. However, traditional concrete production practices rely heavily on natural resources, leading to environmental degradation and resource depletion [1]. Over the past few years, the construction sector has encountered many difficulties related to sustainability and its ecological footprint. The increasing need for construction resources, especially sand, has raised concerns about their depletion and the harmful consequences of sand extraction on the environment [2]. To address these problems, scientists and engineers have investigated alternative materials and approaches to advancing resource efficiency and sustainable waste management [3]. However, sustainable development in the construction plan can be realized only by numerous innovative tactics, such as selecting suitable materials, modern procedures for eco-efficient operations, and waste recycling and reuse. The use of waste materials in concrete is a promising method for managing industrial by-products in an environmentally responsible way while lowering the demand for natural resources [4,5]. One promising approach involves the incorporation of waste materials into concrete production processes.

While previous research has explored using experimental and machine learning algorithms to predict the strength properties of concrete containing waste materials, a significant gap remains in validating and optimizing these predictions. To bridge this gap, comprehensive cost analysis and the application of small dataset ranking score indices through ML algorithms are needed. Additionally, the existing studies often lack a detailed comparative analysis of the performance of different machine learning models tailored explicitly to small datasets, which are common in experimental research involving sustainable materials such as WIS. This gap must be addressed because accurate prediction models considering both performance and cost are crucial for implementing waste iron slag in concrete production. Furthermore, a detailed cost analysis is essential to ensure that the incorporation of waste iron slag is technically viable and economically feasible, encouraging wider adoption of this sustainable practice.

Numerous studies have explored the reuse of various waste materials as partial replacements for fine aggregates in concrete production, including waste foundry sand [6,7], copper slag [8,9], imperial smelting furnace slag [10,11], and blast furnace slag [12,13]. These studies have demonstrated that these materials can be used as partial or complete substitutes for sand in concrete mixtures, with properties comparable to those of control concrete. For example, incorporating up to 20 % foundry sand from the aluminum casting industry closely matched the strength of control mix concrete [14]. However, increased water absorption and decreased compressive strength were reported with higher foundry sand content [15]. Similarly, effective use of foundry sand in ready-mix concrete was observed, provided substitutions did not exceed 20 % to avoid adverse effects.

In the context of steel slag, better resistance to hydrochloric acid than to sulfuric acid in concrete containing steel slag was noted [16], whereas steel slag was reported to negatively affect workability, particularly with substitutions greater than 50 % [17]. The optimal compressive strength was achieved with 15–30 % steel slag substitution. A decrease in compressive strength was reported when more than 30 % induction furnace steel slag was used [18]. Copper slag has also been evaluated as a sand substitute, with no significant changes in column failure load reported with up to 40 % copper slag substitution [19], and up to 40 % copper slag-producing high-strength concrete comparable to or exceeding control mixtures.

Further research on self-compacting concrete (SCC) incorporating iron slag revealed improvements in strength and durability with up to 40 % iron slag replacement [20]. Challenges in recycling scrap iron due to variations in quality were highlighted [21], although studies indicate that carefully managed scrap iron can enhance concrete properties and sustainability [22,23]. Replacing 25–30 % of sand with iron slag significantly increased compressive strength, with the optimum value at 30 % replacement [24].

ML has also been increasingly utilized to predict the strength of concrete made with waste materials, providing a more efficient and precise alternative to traditional experimental methods [[25], [26], [27]]. The random forest (RF) model was identified as the most effective for predicting compressive strength in concrete with iron waste, achieving a coefficient of determination (R^2^) of 0.972 and highlighting key factors such as iron waste, fine aggregate ratios, and concrete age [28]. Similarly, the effectiveness of ML models such as SVR, DT, and the AdaBoost regressor in predicting concrete properties with waste foundry sand was demonstrated, with their SVR-GWO hybrid model achieving near-perfect accuracy (R^2^ = 0.999 for compressive strength and elastic modulus and 0.998 for split tensile strength) [27].

ML models were applied to predict the compressive strength of fiber-reinforced rubberized recycled aggregate concrete (FR3C), with CatBoost identified as the most accurate model, achieving the highest R^2^ and the lowest root mean square error (RMSE) and mean absolute error (MAE) [29]. Their analysis highlighted the water/cement ratio, nominal aggregate size, and rubber percentage as key factors influencing the compressive strength of FR3C. Additionally, response surface methodology (RSM) and artificial neural networks (ANN) were used to predict the compressive strength of glass fiber mortars, with the ANN model proving more accurate than the RSM, particularly for the optimal 0.6 % glass fiber proportion, demonstrating a greater correlation between experimental and predicted results [30]. Another study employed Artificial Neural Networks (ANN), Linear and Non-Linear Multivariate Adaptive Regression Splines (MARS-L and MARS-C), Gaussian Process Regression (GPR), and Minimax Probability Machine Regression (MPMR). The predicted outputs of these conventional machine learning (CML) models were then combined and trained using ANN. This approach was used to develop a Hybrid Ensemble Model (HENSM), which demonstrated significantly improved results compared to the standalone CML models [31]. Again, ANN has been utilized to predict the unconfined compressive strength of granite using only two non-destructive tests, which provide results of weak to very strong granite with a deviation of less than 20 % compared to the experimental data [32]. Another study demonstrated the use of six models—linear regression (LR), multilinear regression (MLR), non-linear regression (NLR), pure quadratic (PQ), interaction (IA), and full quadratic (FQ)—to predict the compressive strength of concrete modified with nanosilica, achieving a promising level of accuracy in strength prediction [33]. Recent research has demonstrated that ensemble convolution-based deep learning algorithms, such as the separate stacking model (SSM) with AdaBoost meta-learner, can effectively estimate the bond strength of corroded reinforced concrete by incorporating key influencing factors, including corrosion level, concrete compressive strength, and transverse reinforcement ratio, achieving high accuracy and reliability [34]. Recent studies have highlighted the efficiency of the MARS-EBS model in predicting the bearing capacity of geogrid-reinforced stone columns, demonstrating superior simplicity and computational speed compared to SVM and ANN models while achieving high accuracy with an R^2^ value of up to 0.995 for total data [35].

This paper investigates the effect of reusing WIS as a partial substitute for fine aggregate in low-strength concrete production. This study addresses the mentioned gap by conducting an experimental investigation combined with a comparative analysis of various machine learning algorithms to predict the strength properties of WIS concrete. Various machine learning models, including SVM, GPR, RF, GBR, DT, XGBoost, and symbolic regression, are evaluated to identify the most effective prediction strength. Additionally, a detailed statistical ranking index and comparison analysis revealed that the key factors affecting strength are the incorporation ratios of WIS and fine aggregate and the concrete's age. It introduces a ranking score index specifically designed for small datasets, enabling a more nuanced model performance evaluation.

Additionally, the study includes a cost analysis to assess the economic implications of using WIS, ensuring that the predictions are accurate and cost-effective. By integrating these elements, this study provides a comprehensive framework that enhances the reliability and practicality of using machine learning to predict the strength properties of sustainable concrete materials. Through experimental analysis and theoretical modeling, the effects of waste iron incorporation on concrete performance are elucidated, highlighting its feasibility as a sustainable alternative in the construction industry.

## 2 Materials and methods

### 2.1 Materials characterization

This study investigated the mechanical and physical properties of concrete, in which waste iron slag (WIS) was partially substituted for fine aggregates. The primary components of the concrete examined include ordinary portland cement (OPC), aggregates, water, and WIS, focusing on the impact of WIS on concrete performance.

A cementitious material possesses adhesive and cohesive properties that bond inert aggregates into a solid, durable mass with adequate strength [36]. The cement used for this work was OPC (BDS EN 197-1: 2010 CEM I, 52.5 N), produced locally. The specific gravity of the cement tested following ASTM C 150 [37] was 3.10, indicating that it was suitable as a binding material for concrete.

WIS is a significant by-product of the steel industry and is often deposited in domestic waste and landfills. It is generated during the iron and steel production process, either from the conversion of iron to steel in a basic oxygen furnace (BOF) or from the melting of scrap steel in an electric arc furnace (EAF) [38]. A sample of the WIS is used in this study, with a specific gravity of 2.7 using ASTM C 150 [37] standard. The maximum particle size of the waste iron slag was 4.75 mm, and the fineness modulus of the particles, determined according to ASTM C136-84 [39], was 3.31.

**Fig. 1:**
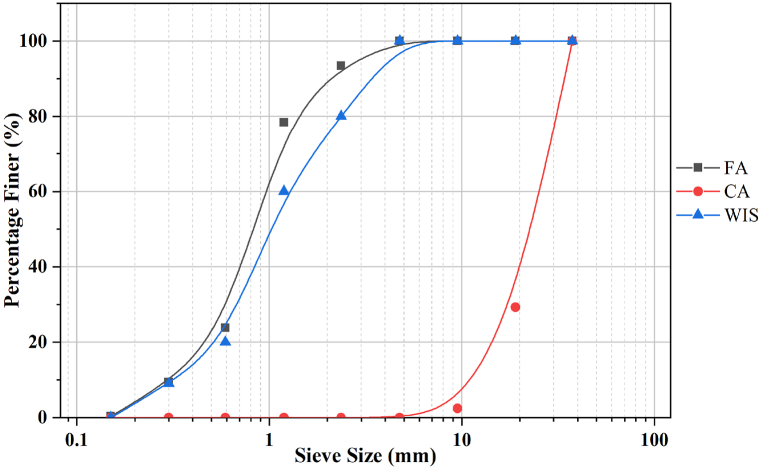
Sieve analysis of fine aggregate (FA), coarse aggregate (CA), and waste iron slag (WIS).

The aggregate selection included natural river sand and crushed gravel, with a maximum nominal size ranging from 12.5 mm to 19 mm. The specific gravity values for the fine and coarse aggregates were 2.55 and 2.77, respectively. Precise particle size distribution curves were established for fine, WIS, and coarse aggregates through manual crushing, aligning with ASTM C136/C136M − 14 (2014) [39] specifications, as depicted in Fig. 1. The maximum fineness modulus values for the fine and coarse aggregates were 2.94 and 6.83, respectively, which are consistent characteristics between the natural coarse and fine aggregates.

Clean potable water was used for both mixing and curing in this study. Water supplied on the IUBAT campus that adhered to potable standards with a pH of 7 was used. The mix design utilized a water-cement (W/C) ratio of 0.45, with the water sourced from Dhaka city's potable supply.

### 2.2 Mix proportions and testing procedure

**Fig. 2:**
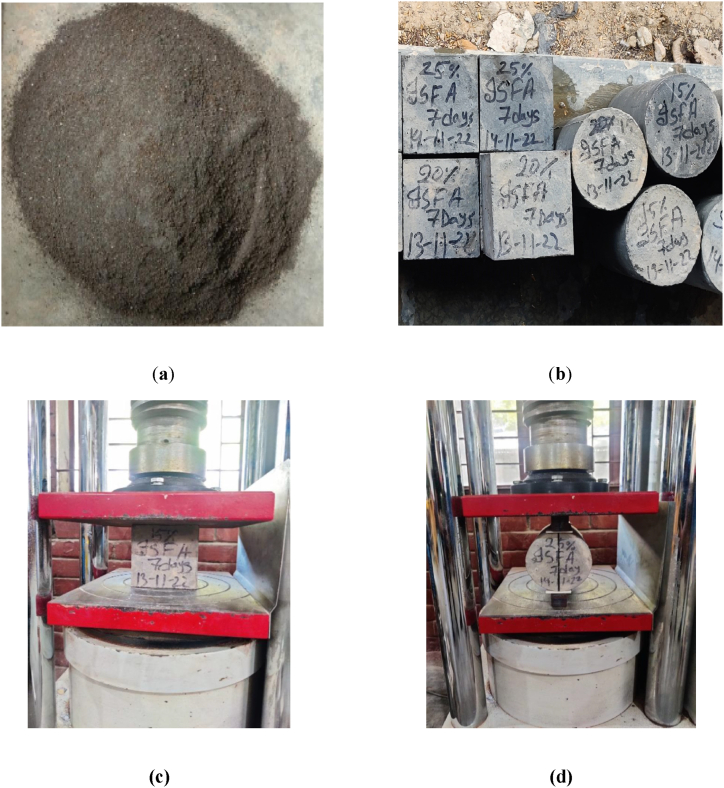
(a) Image of waste iron slag (WIS), (b) sample of concrete, (c) compressive strength test, and (d) splitting tensile strength test.

The material compositions of seven concrete mixtures, with WIS replacing fine aggregates at 0 %, 5 %, 10 %, 15 %, 20 %, 25 %, and 30 % by weight of natural fine aggregate, are detailed in Table 1. Local coarse aggregate and river sand were used for the concrete. The WIS was sourced from local landfill sites and mechanically processed to achieve the desired size, as illustrated in Fig. 2 (a). Before the concrete was mixed, the coarse aggregate was prepared under saturated surface dry (SSD) conditions by soaking it in water for at least 24 h. After soaking, excess water was removed from the aggregate surfaces using a wet towel. It is important to note that the SSD condition was applied only to the coarse aggregate, whereas the fine aggregate was used in an air-dry state.

**Table 1:** Concrete mix proportions used in this study.

**Mix ID**	**Cement (kg/m**^**3**^**)**	**Fine Aggregate (kg/m**^**3**^**)**	**Coarse Aggregate (kg/m**^**3**^**)**	**Waste Iron Slag (kg/m**^**3**^**)**	**W/C**	**Water (kg/m**^**3**^**)**
WIS0	261.8	460.7	933.3	0	0.45	117.8
WIS5	261.8	437.6	933.3	23	0.45	117.8
WIS10	261.8	414.6	933.3	46.1	0.45	117.8
WIS15	261.8	391.6	933.3	69.1	0.45	117.8
WIS20	261.8	368.5	933.3	92.1	0.45	117.8
WIS25	261.8	345.5	933.3	115.2	0.45	117.8
WIS30	261.8	322.5	933.3	138.2	0.45	117.8

Note: WIS means concrete made of waste iron slag.

Immediately after mixing, the workability of the fresh concrete was assessed via a slump test in accordance with ASTM C143 (2014) [40]. All molds were thoroughly cleaned, brushed, and oiled before casting. The molds were filled with three layers. After 6 h, the samples were labeled with their designated identifiers and left to set in the molds for 24 h, as shown in Fig. 2 (b). A minimum of four samples for each mixture and age, totaling 112 samples, were prepared for testing at curing ages of 7 and 28 days. All the samples were cured in water tanks at a temperature of 25 ± 2 °C until the day of testing to ensure a consistent wetting process.

A Universal Testing Machine (UTM) with a 2000 kN capacity was utilized for the mechanical strength tests. A total of 56 concrete cube samples, each measuring 100 mm × 100 mm × 100 mm, were prepared to assess the compressive strength by following BS EN 12390-3 [41] standards, as depicted in Fig. 2 (c). Additionally, 56 cylindrical samples, with a diameter of 100 mm and a length of 200 mm, were used for the splitting tensile strength tests, following ASTM C496 [42]. For the tensile strength test, each cylinder was positioned horizontally between two iron plates—one on top and the other on the bottom—ensuring that the applied force created a line load, as shown in Fig. 2 (d). The load was applied until the sample failed, and the resulting data were recorded. The final compressive and tensile strengths for each mix were determined by averaging the results from four samples.

A single-factor analysis of variance (ANOVA) was conducted to assess the statistical significance of different percentages of WIS on the compressive strength and split tensile strength of the concrete samples. A p-value of 0.05 was used as the threshold for determining the level of significance in the hypothesis test.

### 2.3 Machine learning modeling strategy

In the realm of civil engineering, the application of ML techniques to predict the compressive strength of concrete datasets has gained prominence because of its potentiality to increase both efficiency and accuracy [43,44]. ML strategies such as dimensionality reduction and ensemble methods are invaluable when dealing with limited data; however, ML strategies such as dimensionality reduction and ensemble methods prove invaluable [45].

Normalization is often used in machine learning to scale input features to a similar range, especially when features have varying units or magnitudes, to ensure fair contribution to the model's learning process [46]. However, in this work, normalization was not applied because all input variables are expressed in the same units and scale. When the input features share consistent units, their relative magnitudes inherently represent meaningful relationships within the data, eliminating the need for additional scaling. In this study, all input parameters have comparable ranges and units, and the machine learning algorithms can process these values directly without bias toward features with larger absolute values. Algorithms like RF and DT are particularly robust to unnormalized data in such cases, as they split features based on relative ordering rather than absolute magnitudes. Therefore, normalization was deemed unnecessary for this dataset, simplifying the preprocessing pipeline while maintaining model accuracy.

However, traditional machine learning models face challenges such as overfitting and limited generalizability when dealing with small datasets [47]. Several strategies were implemented to mitigate overfitting in the proposed models. Cross-validation was employed during model training to ensure the generalizability of the models to unseen data. For ensemble models such as RF and Gradient Boosting, regularization techniques, including limiting tree depth, were utilized to control model complexity and prevent overfitting. In models like SVM and XGBoost, default hyperparameters were selected to balance bias and variance, ensuring robust predictive performance while avoiding overfitting. Excessively deep models were avoided, and an appropriate train-test split ratio of 70:30 was maintained to reduce overfitting risks further. These combined measures enhance the models’ robustness, ensuring reliable predictions and maintaining generalizability across datasets. Small data scenarios are common in specialized or early-stage research, where gathering large amounts of data may not be feasible. The main reasons for the small amount of data used in concrete strength prediction include the high cost and time required for experimental testing, the specificity of certain concrete compositions, and the limited availability of historical data. Additionally, some research has focused on innovative materials, leading to inherently smaller datasets [48]. The critical problems associated with small-data machine learning include overfitting, where models learn noise rather than the signal, leading to poor performance on unseen data and less accurate predictions [49].

Here, an overview of how each algorithm applies to small datasets is provided for this study.

Support Vector Machines (SVMs): SVMs are well suited for small datasets because they focus on finding the optimal hyperplane that maximizes the margin between different classes. By using techniques such as kernel tricks, SVMs can effectively handle non-linear relationships in the data [50].

Gaussian Process Regression (GPR): GPR is a nonparametric, probabilistic model that provides predictions with uncertainty estimates. It is particularly effective for small datasets because it does not assume a fixed model structure but instead learns from the data directly [51].

Random Forest (RF): RF is an ensemble method that builds multiple decision trees on different subsets of the data. By aggregating the results, the RF reduces the risk of overfitting, which is a common challenge with small datasets [52].

Gradient Boosting Regression (GBR): GBR builds models sequentially, with each new model attempting to correct the errors of the previous models. It is particularly effective for small datasets because it can adapt to the complexity of the data incrementally, making it robust against overfitting [53].

Decision Trees (DTs): DTs are simple yet powerful models that effectively handle small datasets. However, they are prone to overfitting. To mitigate this, techniques such as pruning and setting a minimum number of samples per leaf can be applied [54].

XGBoost: XGBoost is an optimized gradient-boosting implementation that is faster and more efficient. It can be particularly useful for small datasets as it includes regularization techniques to prevent overfitting, thus improving generalizability [55].

Symbolic Regression: This approach attempts to find mathematical expressions that best fit the data. It is especially useful in small datasets as it can uncover the underlying relationships without assuming a specific model form [56].

In a machine learning framework for this study, the dataset is split into 70 % for training and 30 % for testing various algorithms, such as SVM, GPR, RF, GBR, DT, XGBoost, and symbolic regression, utilize the training subset to build and fine-tune models by optimizing specific parameters such as kernel functions, regularization terms, and tree depth. These algorithms focus on minimizing errors and overfitting while enhancing model accuracy. Each of these algorithms can be effective for small datasets with careful parameter tuning.

The hyperparameters for this study soft computing models were initially selected using default values from their respective libraries, providing a reliable baseline for model evaluation. These defaults were chosen to balance simplicity and performance, allowing the models to demonstrate their inherent capabilities without extensive fine-tuning [57]. For instance, the defaults for SVM, GPR, and XGBoost incorporate configurations that address model complexity and non-linearity effectively, while ensemble methods like RF and GBR leverage settings that reduce overfitting risks. This approach ensures fair comparisons between models and establishes a foundation for further performance enhancements through hyperparameter optimization techniques, such as grid search or random search.

The hyperparameters for the soft computing models were initially chosen based on the default configurations provided by the respective libraries, serving as a baseline for performance evaluation. For Support Vector Machines (SVM), the default Radial Basis Function (RBF) kernel was employed with *C* = 1.0 and ϵ = 0.1, balancing model complexity and error tolerance. The Gaussian Process Regression (GPR) utilized the default RBF kernel with *α* = 1 × 10^−2^, offering regularization to capture non-linear relationships effectively. For the Random Forest (RF) model, *n*_*estimators* = 100 and unrestricted tree depth were applied, ensuring model flexibility while mitigating overfitting. Gradient Boosting Regression (GBR) adopted *n*_*estimators* = 100, *learning*_*rate* = 0.1 and *max*_*dept*ℎ = 3, providing a balance between performance and overfitting control. The Decision Tree (DT) model was configured with *max*_*dept*ℎ = *None* and *min*_*samples*_*split* = 2, enabling comprehensive growth for pattern identification. For XGBoost, *n*_*estimators* = 100, *learning*_*rate* = 0.1, and *max*_*dept*ℎ = 3 were selected to optimize performance while maintaining simplicity. Symbolic Regression (SR) applied evolutionary techniques with a default configuration of *population*_*size* = 1000, *generations* = 20, and *tournament*_*size* = 20, ensuring effective genetic algorithm-based optimization. While these defaults provided a robust baseline, future enhancements, such as hyperparameter tuning through grid search or other optimization techniques, could further refine model performance and generalizability.

#### 2.3.1 Validation methods for prediction models

In assessing machine learning models, particularly for regression tasks, various performance metrics are utilized to understand the model's accuracy and reliability fully. The coefficient of determination (R^2^) is a key metric that indicates the proportion of variance in the dependent variable that can be explained by the independent variables. An R^2^ value closer to 1 suggests a strong correlation between the model's predictions and actual outcomes, implying that the model captures most of the data's variability [58]. However, R^2^ alone may not always provide a complete assessment, especially when dealing with non-linear relationships or models with many predictors.

The root mean square error (RMSE) is another critical metric, particularly valued for its ability to penalize large errors more than smaller ones, which measures how close the predictions are to the actual results [59].

The mean absolute error (MAE), on the other hand, gives a straightforward average of absolute errors, offering an easy-to-interpret measure of model performance. Unlike RMSE, MAE treats all errors equally, which can be advantageous in scenarios where all deviations, regardless of magnitude, are equally important [60].

The scatter index (SI) is a normalized metric often expressed as a percentage and is calculated by dividing the RMSE by means of the observed data. It offers a dimensionless error measure, making it helpful in comparing model performance across different datasets or conditions [61].

The “a20-index” is a simple yet significant statistical measure used to evaluate the performance of predictive models, especially in engineering applications. It represents the proportion of samples for which the model's predictions deviate by no more than ±20 % from the actual experimental values [62].

Combining these metrics enables a more accurate evaluation of model performance, allowing researchers to understand how well their models align with the underlying data. Each metric provides a different lens through which to view the model's predictive accuracy, and together, they offer a robust assessment framework. Equations (1), (2), (3), (4), (5) mathematically express these performance metrics [63,64].

(1) $$R^{2}=1-\frac{\sum_{i=1}^{n}{\left(P_{y}-O\right)}^{2}}{\sum_{i=1}^{n}{\left(O-\bar{O}\right)}^{2}}$$

(2) $$MAE=\frac{1}{n}\sum_{i=1}^{n}\left|\left(P_{y}-O\right)\right|$$

(3) $$RMSE=\sqrt{\frac{1}{n}{\sum_{i=1}^{n}\left[\left(P_{y}-O\right)\right]}^{2}}$$

(4) $$SI=\frac{RMSE}{t^{\prime }}$$

(5) $$a20-index=\frac{m20}{M}$$

where n is the number of observations, p_y_ is the value predicted by the model, o is the observed value, $\bar{o\;}$ is the average of the actual values, and t’ is the mean of the observations at each grid point. According to Equation (5), “M” denotes the total number of samples in the dataset, while “m20” represents the number of samples where the ratio of the “observed value” to the “predicted value”.

## 3 Results and discussion

### 3.1 Experimental part

#### 3.1.1 Slump and bulk density of WIS concrete

**Fig. 3:**
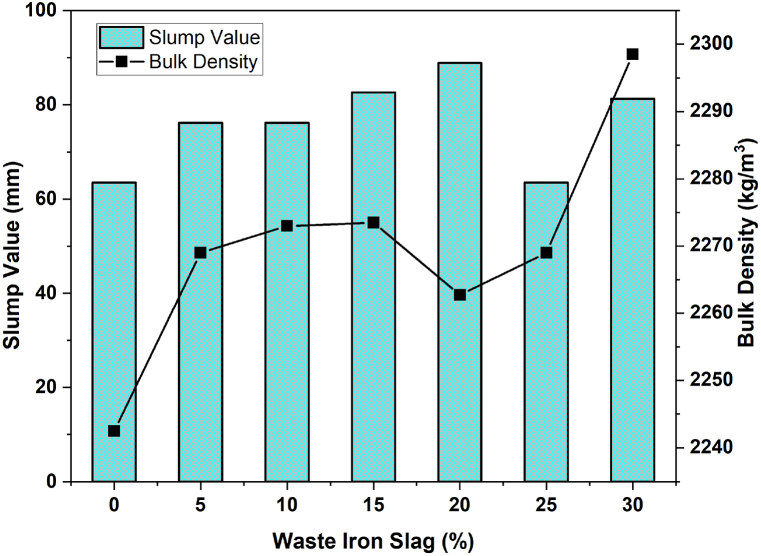
Effects of WIS content on the slump value and bulk density of concrete.

The impact of varying percentages of waste iron slag on a concrete mix's slump value and bulk density is illustrated in Fig. 3. As the percentage of waste iron slag increases from 0 % to 10 %, the slump value slightly increases, suggesting an improvement in workability. However, above 10 %, the slump value exhibited minor fluctuations, decreasing by approximately 20 % before increasing again to 30 %. Concurrently, the bulk density, shown by the line graph, initially increases with up to 10 % slag, implying a denser concrete mix. This trend slightly reversed at approximately 20 %, indicating a reduction in density before increasing significantly at 30 %, where the bulk density reached approximately 2290 kg/m³. These observations suggest that incorporating waste iron slag into concrete influences both its workability and density, with potential implications for optimizing the mix for specific structural requirements. The interplay between these properties highlights the importance of carefully balancing the percentage of slag to achieve the desired concrete characteristics.

The increase in both density and slump value in WIS concrete without additional water is due to the specific properties of WIS, such as smoother texture or different particle shapes, which reduce internal friction and improve workability [65]. Additionally, lower porosity and water absorption of WIS compared to natural aggregates can enhance dispersion, leading to better flow and higher slump values, even as density increases. However, beyond certain levels, the slump value decreases as the mixture may become too stiff or lose cohesion. The bulk density increases when WIS particles effectively fill voids, leading to a denser mix, but it can decrease if WIS causes higher porosity or less efficient packing. These changes in slump and bulk density affect the mechanical properties of WIS concrete. A higher slump can improve workability but may reduce strength if excessive, whereas increased bulk density typically enhances compressive strength by reducing the number of voids. Conversely, a lower bulk density can weaken the concrete, reducing durability and strength. Therefore, balancing the WIS content is crucial for optimizing both the workability and strength of a concrete mixture.

#### 3.1.2 Compressive and split tensile strengths of WIS concrete

**Fig. 4:**
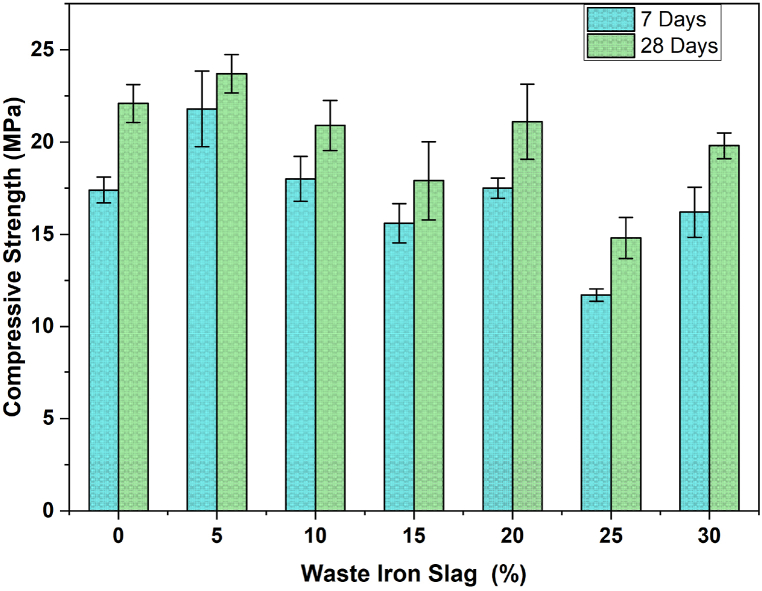
Compressive strength variation with waste iron slag percentage over time.

The compressive strength of the concrete at 7 and 28 days with varying percentages of WIS from 0 % to 30 % is shown in Fig. 4. Initially, at 0 % WIS (measured), the concrete achieves its highest strength, reaching approximately 22 MPa after 28 days. The observed CS of 22 MPa, compared to the expected 38 MPa for a 0.45 w/c ratio as per ACI 211 [66], results from several factors, including lower quality cement or aggregates, improper mixing, and curing, or testing inaccuracies. With 5 % WIS, the strength remains relatively high; however, as the WIS content increases from 10 % to 20 %, a noticeable decline in compressive strength indicates that higher WIS percentages adversely affect the structural integrity of the concrete. At 25 % WIS, the strength decreases further, but a slight recovery is observed at 30 %, possibly due to better particle packing or pozzolanic reactions [67] at higher WIS levels. The compressive strength analysis with varying percentages of WIS reveals a general decreasing trend as the WIS content increases. At 0 % WIS, the compressive strength is the highest, with 20 MPa at 7 days and 24 MPa at 28 days. As WIS is introduced, the strength gradually decreases, with a notable decrease observed at 25 % WIS, where the 7-day strength decreases to 11 MPa and the 28-day strength decreases to 16 MPa, representing a significant reduction of approximately 35 % from the control sample (0 % WIS). However, at 30 % WIS, there is a slight recovery in strength, but it remains below the measured, indicating that while small amounts of WIS can be tolerated, higher percentages significantly weaken the concrete. This trend indicates that while small amounts of WIS (up to 5 %) do not severely affect strength, higher percentages can reduce the CS and durability of concrete, making it less suitable for structural applications without careful mix optimization. The compressive strength values of WIS concrete are not linear with increasing WIS percentage because various factors affect the microstructure and overall performance of the concrete. As WIS is introduced, it alters the aggregate composition and particle packing density, leading to inconsistent bonding and variations in strength. Additionally, the chemical interactions between WIS and the cement matrix can vary, contributing to the non-linear strength results. These combined effects result in a complex relationship between the WIS content and compressive strength, where the optimal strength is only achieved at specific WIS levels.

The observed trends in compressive strength with varying percentages of WIS in concrete align with findings from previous studies. The initial decrease in compressive strength with increasing WIS content, as shown in the graph, is consistent with studies that reported similar decreases when iron slag was used as a partial aggregate substitution in self-compacting concrete (SCC) and other concrete types [68]. These studies noted that higher percentages of slag can disrupt the material's binding efficiency, leading to reduced strength. However, the slight recovery in strength at higher WIS percentages, such as at 30 %, suggests that, as in other studies, a more optimized slag content can contribute positively to concrete density and binding properties the density and binding properties of concrete, potentially through improved particle packing. Thus, the results indicate that while WIS can be beneficial at lower percentages, its effects on concrete strength need careful consideration and optimization, echoing the findings of previous studies.

**Fig. 5:**
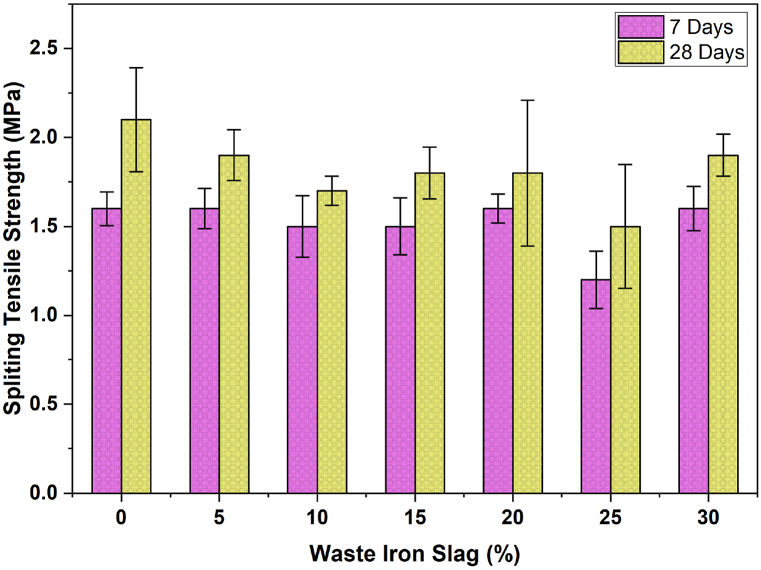
Effect of WIS content on the splitting tensile strength of concrete over time.

In addition, Fig. 5 shows that the splitting tensile strength of concrete at both 7 and 28 days decreases nonlinearly with increasing percentages of waste iron slag (WIS). At 7 days, the strength initially increases by about 10 % at 5 % WIS but then gradually decreases, with a 15 % reduction observed at 25 % WIS, followed by a slight recovery at 30 %, bringing it to approximately 10 % below the 0 % WIS measured. At 28 days, the strength shows minimal change at 5 % WIS but decreases by approximately 10 % at 10 % WIS, continuing to decrease by approximately 20 % at 25 % WIS, with a minor recovery at 30 %, still about 10 % below the measured. These results highlight the complex impact of WIS on tensile strength, with varying reductions and slight recoveries at different slag levels, indicating the need for careful optimization of the WIS content in concrete mixtures. The initial decrease in tensile strength with increasing WIS content can be attributed to the disruption of the concrete matrix due to the introduction of slag, which may not bond as effectively as traditional aggregates do. The slight recovery in strength at higher percentages, particularly at 20 % and 30 %, could be due to improved particle packing or pozzolanic activity of the slag, contributing to better tensile properties. Overall, the variation in the splitting tensile strength with different WIS percentages indicates that while small amounts of WIS may reduce the tensile strength, the optimal WIS content still enhance the specific mechanical properties of the concrete, making it necessary to balance WIS content carefully to achieve the desired outcomes.

Overall, while the current results broadly agree with previous research showing an initial decrease in tensile strength with increasing WIS content, the slight recoveries observed at higher percentages highlight the potential for optimizing WIS content to achieve better mechanical properties in concrete. This suggests that the impact of WIS on tensile strength is complex and context-dependent, requiring careful balance to leverage the benefits while minimizing potential downsides.

#### 3.1.3 Effects of different waste fine aggregates on the concrete strength

**Fig. 6:**
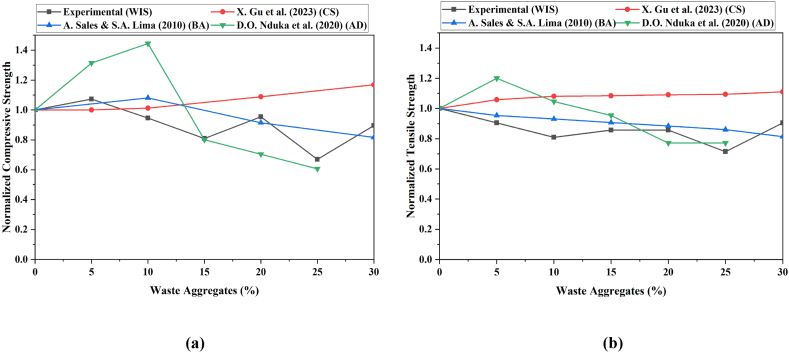
Effects of different waste fine aggregates on the (a) compressive strength and (b) split tensile strength of concrete.

Fig. 6(a and b) compares the normalized compressive and tensile strengths of concrete by using various waste fine aggregates, including WIS, with the results of other researchers. The experimental data show an initial decrease in compressive and tensile strength as the percentage of WIS increases, stabilizing at approximately 20 % content. In contrast, the findings indicate a more consistent performance, with normalized strengths remaining stable across all aggregate percentages, suggesting that their chosen copper slag (CS) waste material, likely crushed stone, integrates more effectively with the concrete matrix [69]. A recent study reported fluctuating strengths, particularly tensile strength, with a peak at approximately 5 %–10 % before declining, indicating that their aggregate (brick aggregate) initial strength benefits but becomes less effective at higher percentages of using bagasse ash (BA) [70]. Similarly, significant strength variations, particularly in compressive strength, show a notable decrease at lower aggregate percentages, stabilizing at higher percentages utilizing aluminum dross (AD) concrete [71]. This variability may result from the different aggregates used, such as ceramic and demolition debris, which have distinct effects on the properties of the concrete.

These comparisons suggest that the type of waste aggregate significantly influences the concrete strength, with some materials offering better compatibility and performance than others. The non-linear trends observed across different studies underscore the importance of selecting the appropriate type and percentage of waste aggregates to optimize the mechanical properties of concrete.

#### 3.1.4 Relationship between strength and percentage of waste aggregate

**Fig. 7:**
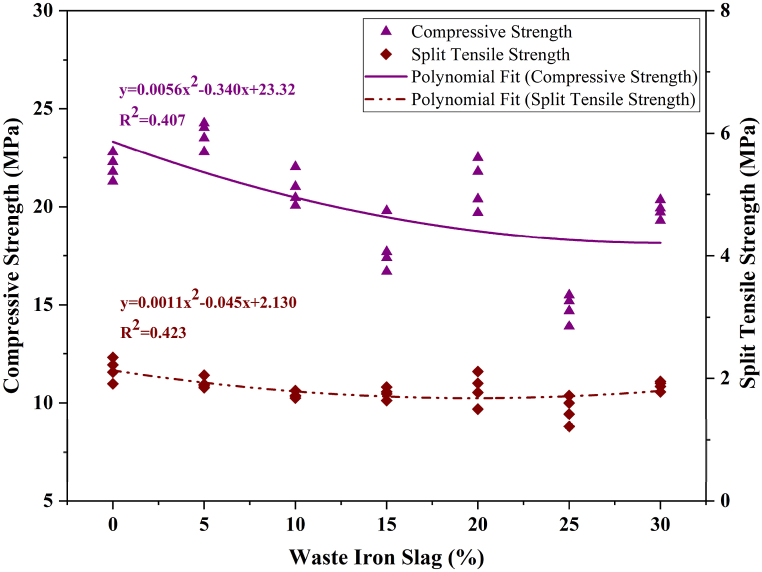
Effect of WIS percentage on compressive and split tensile strengths with polynomial fits.

Fig. 7 shows the relationship between concrete's compressive and split tensile strengths of concrete with varying percentages of WIS. As the WIS content increases, both the compressive and tensile strengths generally decrease. The polynomial fit equations show that the relationship between the WIS percentage and both strengths is non-linear, with R^2^ values of 0.407 for the compressive strength and 0.423 for the split tensile strength, indicating a moderate fit. These relatively low R^2^ values suggest that the WIS percentage alone does not fully explain the variations in strength, likely because of other influencing factors such as the microstructure of the concrete, the mixing quality, and the interaction between the WIS and other concrete components.

The lower R^2^ values indicate significant variances in the data not accounted for by the WIS percentage, implying that the relationship between WIS content and concrete strength is complex and influenced by multiple variables beyond just the percentage of WIS. These factors could include the quality of WIS, its particle size distribution, or other additives in the mixture.

Future studies should consider incorporating more variables, such as the water-to-cement ratio, curing conditions, and specific characteristics of the WIS used, into the analysis to improve the results. Additionally, exploring different combinations of WIS with other supplementary materials might yield a more accurate prediction model, leading to a higher R^2^ value and a better understanding of the strength characteristics of WIS concrete.

The moderate R^2^ values in the polynomial models presented in this study suggest that additional factors influencing strength are not fully accounted for by the current regression models. ML algorithms, which are known for handling small datasets and modeling complex relationships, could offer more accurate predictions by incorporating a broader range of input variables and their interactions. Integrating ML into this study could be both valid and beneficial, as these algorithms could be trained on experimental data to more precisely predict compressive and tensile strengths, potentially enhancing R^2^ values and providing a better fit to the observed data.

#### 3.1.5 Prediction of the modulus of elasticity (MoE)

**Fig. 8:**
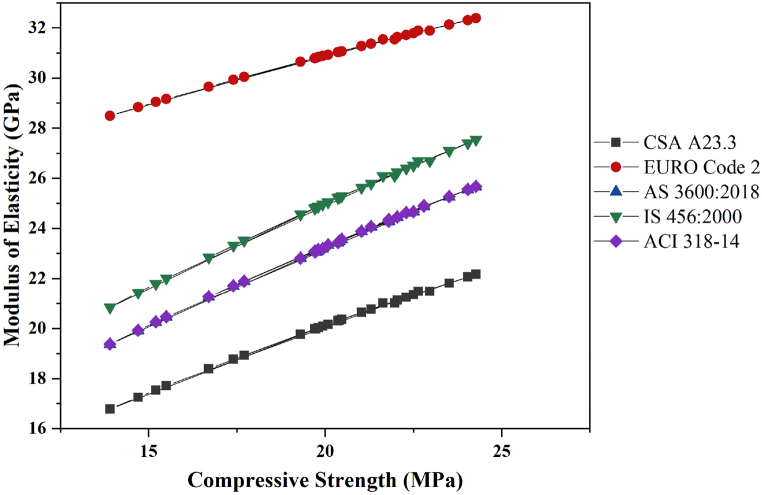
Comparative analysis of the elastic modulus and compressive strength.

A comparative analysis of the MoE (in GPa) versus compressive strength (in MPa) of concrete, as predicted by various international standards and codes, including CSA A23.3, EURO Code 2, AS 3600:2018, IS 456:2000, and ACI 318-14, is presented in Fig. 8. The MoE for standard concrete generally ranges from 15 to 24 GPa, depending on the compressive strength and mix composition [72]. These datasets likely correspond to different types of concrete mixtures or materials, as indicated by the varying standards or codes (such as CSA A23.3-14, EURO Code 2, AS 3600:2018, IS 456:2000 and ACI 318-14) used to determine the modulus of elasticity values. The following overview includes the relevant Equations (6), (7), (8), (9), (10) used in these standards [73].

(6) $$E_{C}=4.5\sqrt{f_{c}^{\prime }}\;\left(\text{CSA}\;A23.3-14\right)$$

(7) Ecm=22α(fcm′10)13(EUROCode2)

where, $f_{cm}^{\prime }=f_{c}^{\prime }+8$ MPa and $f_{c}^{\prime }$ is the cube compressive strength of the concrete, and the coefficient α is 0.9 for limestone.

(8) $$E_{C}=\rho_{c}^{1.5}\left(0.043\sqrt{f_{cmi}^{\prime }}\right)\times 10^{-3}\left(\text{AS}\;3600:2018\right)$$

(when $f_{c}^{\prime }$ ≤ 40 MPa).

where, $f_{cmi}^{\prime }$ is the mean value of the in-situ compressive strength of the concrete at the relevant age and $\rho_{c}$ is the unit weight of the concrete.

(9) $$E_{C}=5.59\sqrt{f_{c}^{\prime }}\;\left(\text{IS}\;456:2000\right)$$

(10) $$E_{C}=0.56\sqrt{f_{c}^{\prime }}\;\left(\text{ACI}\;318-14\right)$$

According to the EURO Code 2, the predicted MoE for a given compressive strength indicates that concrete designed under this standard is expected to exhibit greater stiffness. AS 3600:2018 and IS 456:2000 offer slightly lower predictions, still suggesting relatively high stiffness. In contrast, ACI 318-14 forecasts a lower modulus of elasticity, implying that concrete under their standard is less stiff than those governed by EURO Code 2. Finally, CSA A23.3 has the lowest modulus of elasticity, suggesting that concrete following this standard is expected to be the least stiff among the standards.

This variation highlights the importance of selecting the appropriate standard on the basis of regional practices, material properties, and specific project requirements. Depending on the standard chosen, the predicted performance of the concrete can vary significantly, affecting design decisions and safety margins. The application of these findings is crucial when waste iron slag is used in concrete, as the choice of standard affects the estimated stiffness and, consequently, the structural performance of slag-containing concrete.

#### 3.1.6 ANOVA results of WIS concrete properties

A single-factor ANOVA test was conducted at the 95 % significance level to evaluate the statistical relationships among various concrete mixtures on the basis of properties such as compressive strength and split tensile strength. According to the data presented in Table 2, the p-value is less than 0.05 for the split tensile strength, except for the compressive strength evaluation. This finding indicates that there is no statistically significant relationship between the WIS content and split tensile strength. However, a statistically significant relationship exists between the WIS content and compressive strength. Consequently, increasing the proportion of WIS did not significantly alter the compressive strength of the concrete compared with that of the control mix. These findings suggest that WIS can be effectively used as a partial replacement for fine aggregates without compromising the compressive strength of concrete. This conclusion underscores the potential of using WIS in concrete production, contributing to sustainability efforts by reducing waste and conserving natural resources while maintaining the structural integrity of the material.

**Table 2:** ANOVA test results for different concrete mixtures and properties.

**Groups**	**Source of Variation**	**Degree of Freedom**	**Sum of Squares**	**Mean** **Square**	**F Test**	**p Value**	**Significance**
WIS (%) to compressive strength	Between Groups	1	89.006	89.006	1.421	0.256	Yes
	Within Groups	12	751.59	62.633			
WIS (%) to split tensile strength	Between Groups	1	608.520	608.52	10.428	0.007	No
	Within Groups	12	700.208	58.350			

### 3.2 Machine learning algorithm implementation

#### 3.2.1 Performance evaluation of the algorithms on WIS concrete

**Fig. 9:**
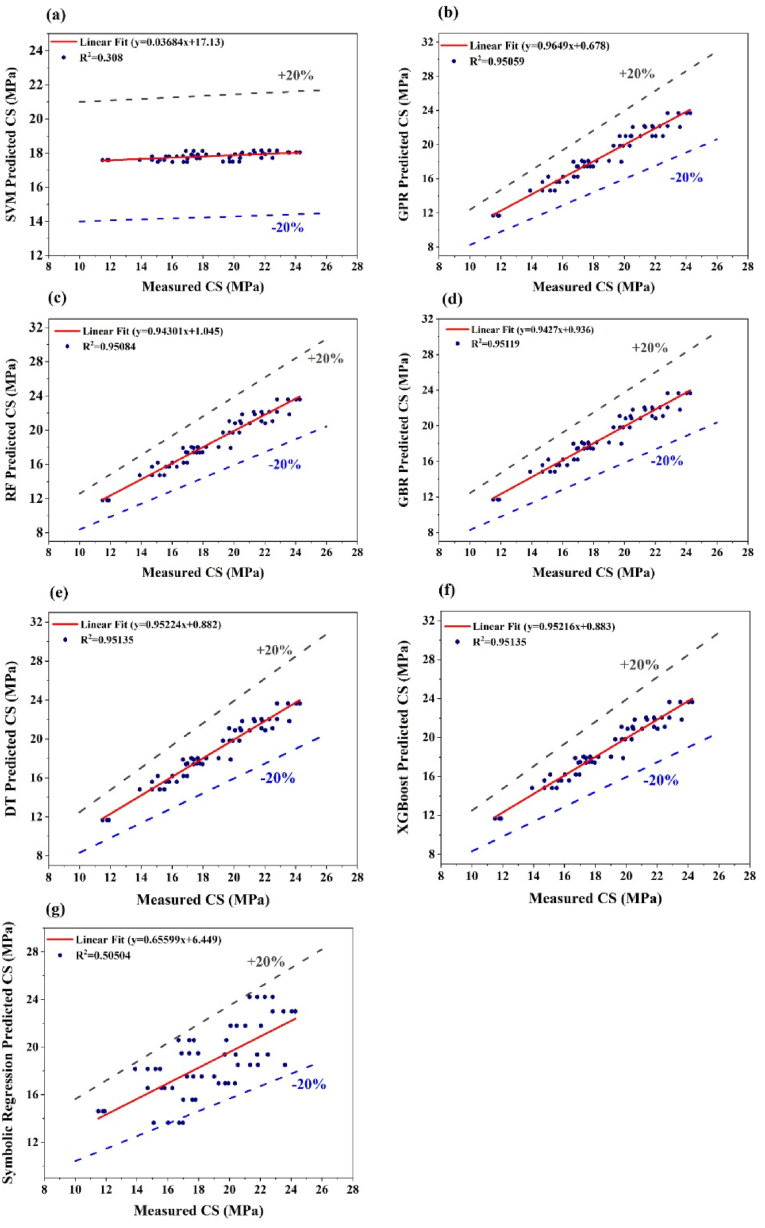
Regression plots of the ML model for compressive strength (CS), (a) SVM algorithm, (b) GPR algorithm, (c) RF algorithm, (d) GBR algorithm, (e) DT algorithm, (f) XGBoost algorithm, and (g) symbolic regression algorithm.

At this stage, the performance of each ML method in estimating the concrete compressive strength is examined and compared with that of laboratory tests. The regression plots in Fig. 9(a–g) present the performance of various machine learning algorithms in predicting the CS of concrete samples. Each plot compares the predicted CS values with the actual measured CS values. The SVM algorithm exhibits the weakest performance, with a significant spread in the data points and an R^2^ value of 0.3086, indicating low predictive accuracy. In contrast, the GPR algorithm shows a much tighter grouping of data points along the line of best fit, yielding an R^2^ value of 0.9505, which signifies high accuracy. The RF and GBR algorithms perform similarly to GPR, with R^2^ values of 0.95084 and 0.95119, respectively, reflecting strong predictive capabilities. The DT and XGBoost models stand out as the top performers, achieving the highest R^2^ value of 0.95135, indicating that both achieve the highest R^2^ value of 0.95135, indicating a nearly perfect correlation between the predicted and measured values. In addition, the symbolic regression algorithm shows a noticeable decline in performance compared with the other models, with scattered data points and an R^2^ value of 0.50504. This places it above the SVM but well below the other algorithms in terms of predictive accuracy. Overall, while the DT and XGBoost models provide the most accurate predictions for concrete compressive strength, closely followed by the RF, GBR, and GPR models, the SVM and Symbolic Regression models exhibit significantly lower predictive accuracy, with the SVM being the least effective. The comparison highlights the variability in model performance, with DT and XGBoost emerging as the most reliable algorithms for this specific application.

The poor performance of the SVM model in predicting concrete compressive strength can be attributed to its limitations in capturing complex, non-linear relationships within the data. SVM is a powerful tool for classification and regression, but it relies on finding a hyperplane that best separates the data points, which may not be sufficient when the underlying relationship between features and the target variable is highly non-linear or involves intricate interactions. If the data are noisy or if the model parameters, such as the kernel type and regularization, are not optimally tuned, the SVM may fail to capture the true patterns, leading to lower predictive accuracy and weaker correlations, as reflected in the R^2^ value.

In contrast, the strong performance of the DT and XGBoost models is due to their inherent ability to handle complex, non-linear interactions between features. Decision trees (DTs) recursively split the data on the basis of the most significant features, effectively modeling complex relationships without requiring extensive preprocessing. XGBoost, an ensemble method based on boosting, further enhances this capability by iteratively refining the model, correcting errors from previous iterations, and applying regularization to prevent overfitting. This results in a robust model that can accurately predict outcomes even in challenging datasets, leading to high R^2^ values and strong correlations with actual compressive strength measurements observed in the DT and XGBoost models.

Adding the ±20 % margin lines to Fig. 9(a–g) provides a clearer visual framework for assessing prediction accuracy by showing how well the predicted values align with the measured values within an acceptable range of variation [57]. For models like RF, GBR, DT, and XGBoost, the majority of predicted values fall within the ±20 % bounds, demonstrating strong predictive performance supported by high R^2^ values (above 0.95), which indicate a strong correlation between predicted and measured values. In contrast, SVM and Symbolic Regression models exhibit lower R^2^ values (0.308 and 0.505, respectively), with many points lying outside the ±20 % bounds, suggesting less reliable predictions. Models like RF, GBR, and XGBoost achieve high R^2^ values and show a tight clustering of points near the ideal line (y = x), indicating accurate predictions. The ±20 % bounds highlight the robustness of these models, while the performance of SVM and Symbolic Regression indicates the need for improvement for this dataset. This visual enhancement underscores the predictive reliability of RF, GBR, and XGBoost while identifying areas of weakness in other models.

The comparison of the discussed machine learning (ML) algorithms with results from previous studies demonstrates consistent findings regarding their predictive capabilities. The DT and XGBoost models achieve the highest R^2^ value of 0.95135, closely followed by RF, GBR, and GPR models with R^2^ values above 0.95. These models effectively capture non-linear and complex interactions due to their advanced handling of features and regularization techniques, as also confirmed in previous studies that emphasize the robustness of ensemble methods like GBR and RF in similar applications [74]. The SVM and Symbolic Regression algorithms display lower predictive accuracy with R^2^ values of 0.308 and 0.505, respectively. SVM's limitations arise from its inability to model highly non-linear relationships unless optimally tuned adequately. These findings align with prior studies that highlight SVM's struggles with noisy or intricate datasets [75]. Adding ±20 % margin lines provide a clear measure of prediction accuracy, emphasizing that models like DT, XGBoost, RF, and GBR have the most predicted values within the margin, highlighting their reliability. In contrast, the broader spread of SVM and Symbolic Regression predictions indicates inconsistency and poor accuracy [76]. Overall, the comparison highlights XGBoost and DT as the most reliable methods for predicting concrete compressive strength, with RF, GBR, and GPR as robust alternatives. SVM and Symbolic Regression require further refinement to match these levels of accuracy.

**Fig. 10:**
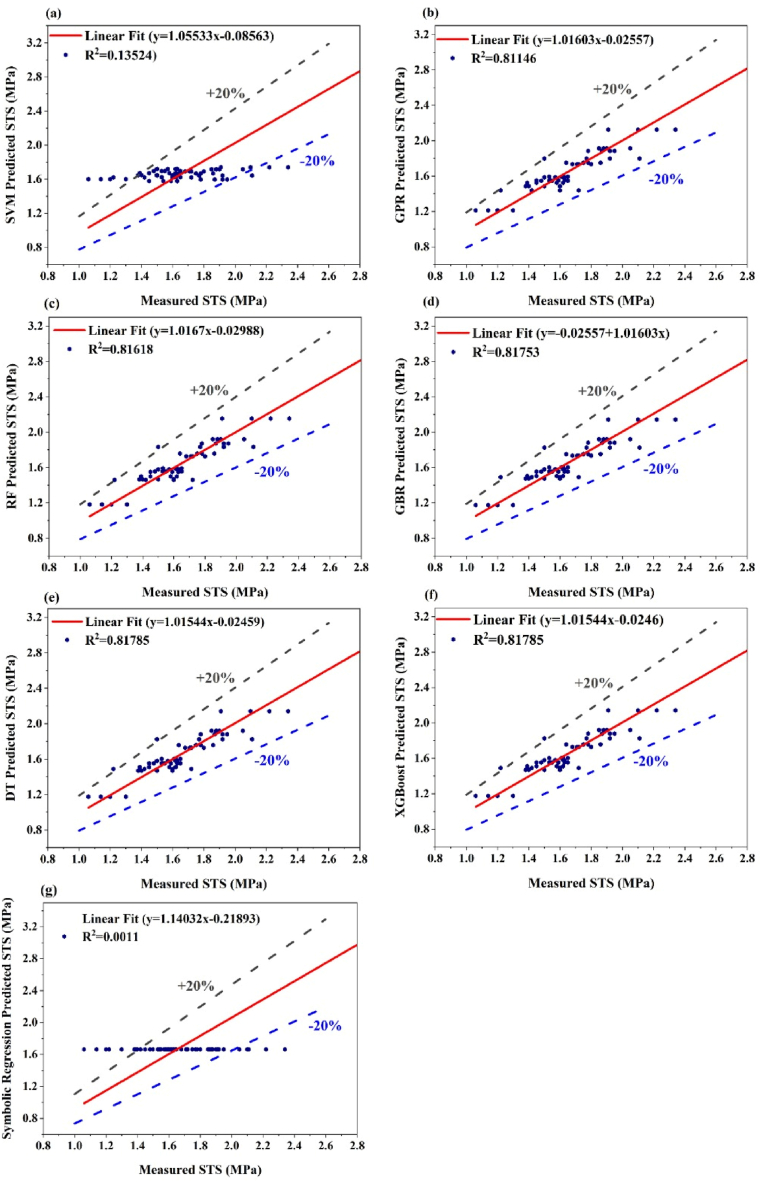
Regression plots of the ML model for split tensile strength (STS) (a) SVM algorithm, (b) GPR algorithm, (c) RF algorithm, (d) GBR algorithm (e) DT algorithm (f) XGBoost algorithm and (g) symbolic regression algorithm.

The regression plots for predicting STS via different ML algorithms highlight the varying levels of performance depicted in Fig. 10(a–g). The SVM model shows a weak correlation, with significant scatter around the regression line and an R^2^ value of 0.13524, indicating low predictive accuracy. In contrast, the GPR model performs better, with data points more closely aligned with the line of best fit, achieving an R^2^ value of 0.81146. The RF and GBR models further improve upon this, showing even tighter correlations with R^2^ values of 0.81618 and 0.81771, respectively, demonstrating the ability of the RF and GBR models to predict stress. The DT and XGBoost models stand out with the highest accuracy, achieving R^2^ values of 0.81785, indicating their effectiveness in modeling the relationships between the input features and the STS.

In contrast, the symbolic regression model significantly underperformed, with an R^2^ value of just 0.0011 and a flat line of predicted values, indicating its inability to capture the variability in the data. The varying performance levels across these models are due to the strengths and weaknesses inherent in each algorithm. SVMs tend to struggle with capturing complex, non-linear relationships, particularly when the data are noisy or not optimally tuned, resulting in poor performance. Conversely, DT and XGBoost are better suited for handling such complexity, with DT using recursive splits on the basis of feature importance and XGBoost employing boosting techniques to iteratively enhance model accuracy iteratively, leading to the strong correlations observed. The poor performance of symbolic regression likely stems from its approach of fitting mathematical expressions to the data, which may not effectively capture the intricate patterns present in the STS dataset.

Additionally, Fig. 10(a–g) illustrates the predicted versus measured STS for various machine learning models, with the addition of ±20 % margin lines offering insight into prediction accuracy. A ±20 % margin is preferred because it provides a more precise range of acceptable deviation between predicted and measured values, ensuring higher accuracy in machine learning models predicting STS. A ±20 % margin ensures precision, aligns with industry standards, and minimizes errors, enhancing the ability to evaluate model performance effectively [26]. Models like GPR, RF, GBR, DT, and XGBoost demonstrate strong linear trends with R^2^ values around 0.81 and most predictions falling within the ±20 % range, signifying reliable performance. These models exhibit minimal bias, as their regression lines closely align with the ideal line. Conversely, the SVM and symbolic regression models show weaker performance, with lower R^2^ values (e.g., 0.135 for SVM and 0.0011 for symbolic regression). This indicates a poor correlation between predictions and actual values, with many points fallings outside the ±20 % limits, reflecting significant over- or under-prediction. The addition of the ±20 % lines provides a clear visual metric to gauge prediction variability and highlights the superior performance of ensemble-based algorithms (e.g., XGBoost) in this context. Overall, the plots emphasize the importance of selecting models with high predictive accuracy and minimal deviation for robust STS prediction.

Ensemble models like DT, XGBoost, RF, and GBR show superior predictive accuracy in predicting splitting tensile strength (STS), with R^2^ values around 0.81785, effectively handling non-linear patterns and complex interactions, aligning with prior research on their robustness in modeling concrete properties [77]. GPR achieved an R^2^ of 0.81146, comparable to ensemble methods, while SVM and Symbolic Regression performed poorly with R^2^ values of 0.13524 and 0.0011, respectively, due to difficulties in handling non-linear and noisy datasets unless carefully tuned, consistent with findings in previous studies on their limitations in regression tasks [78].

#### 3.2.2 ML evaluation for WIS concrete via a statistical scoring index

Table 3 presents a detailed assessment of various ML algorithms in predicting concrete properties, specifically CS and STS, based on several statistical metrics. The algorithms are ranked according to their performance across metrics such as R^2^, MAE, RMSE, SI, and a20-index, with lower ranking scores indicating higher accuracy and higher scores reflecting lower accuracy.

**Table 3:** Evaluating ML algorithms for WIS concrete via statistical scoring index.

Evaluation Criteria/ML Algorithms	**SVM**	**GPR**	**RF**	**GBR**	**DT**	**XGBoost**	**Symbolic Regression**
R^2^ (CS)	0.309	0.951	0.951	0.951	0.951	0.951	0.505
RS (CS)	6	4	3	2	1	1	5
MAE (CS)	2.617	0.569	0.559	0.557	0.553	0.553	2.069
RS (CS)	7	5	4	3	2	1	6
RMSE (CS)	3.205	0.719	0.715	0.712	0.711	0.711	2.360
RS (CS)	7	4	3	2	1	1	5
SI (CS)	0.174	0.039	0.039	0.039	0.039	0.039	0.128
RS (CS)	7	4	3	2	1	1	5
a20-index (CS)	0.768	1.000	1.000	1.000	1.000	1.000	0.857
RS (CS)	3	1	1	1	1	1	2
Total RS (CS)	30	18	14	10	6	5	23
R^2^ (STS)	0.135	0.811	0.816	0.818	0.818	0.818	0.001
RS (STS)	6	5	4	3	1	1	7
MAE (STS)	0.186	0.083	0.083	0.082	0.082	0.082	0.198
RS (STS)	6	5	4	3	1	2	7
RMSE (STS)	0.237	0.111	0.109	0.109	0.109	0.109	0.255
RS (STS)	6	5	4	3	1	2	7
SI (STS)	14.327	6.690	6.605	6.578	6.575	6.575	15.410
RS (STS)	6	5	4	3	1	1	7
a20-index (STS)	0.830	1.000	0.982	0.964	0.964	0.964	0.821
RS (STS)	4	1	2	3	3	3	5
Total RS (STS)	28	21	18	15	7	9	33

∗RS=Ranking score, CS=Compressive strength, STS=Split tensile strength.

**Fig. 11:**
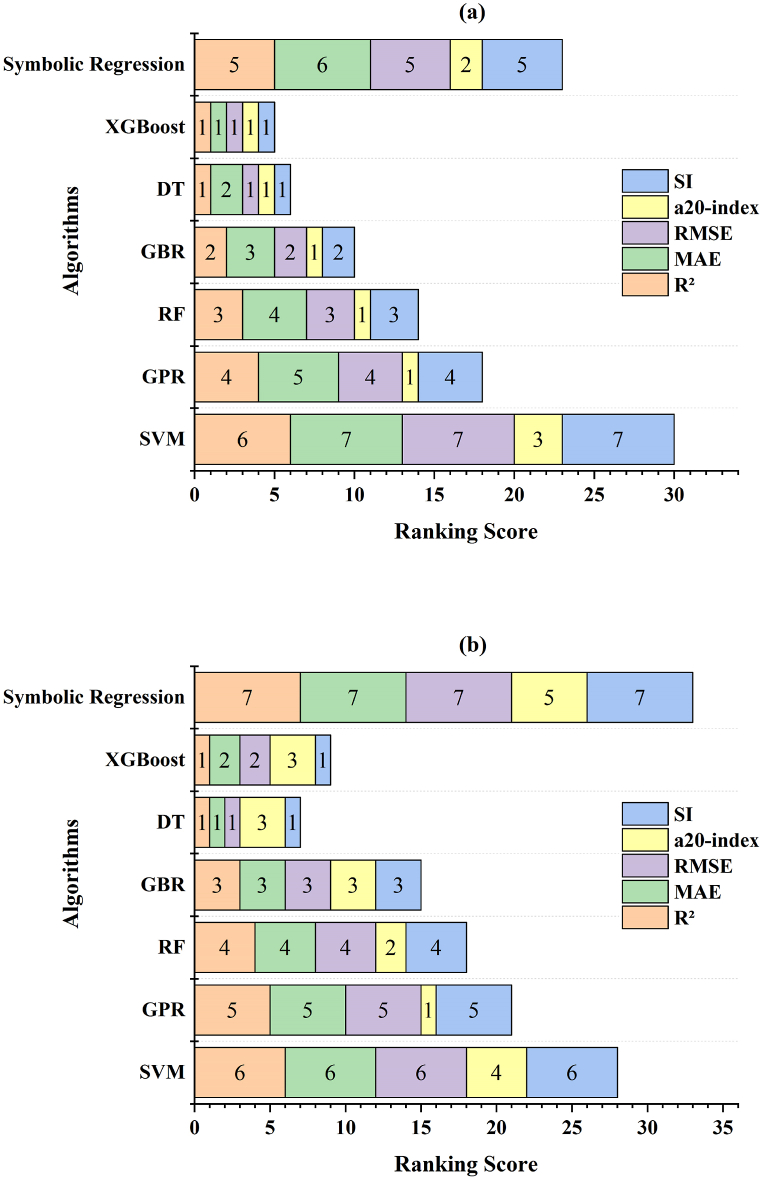
Ranking score of the ML algorithms for (a) compressive strength and (b) split tensile strength.

For the CS in Fig. 11 (a), the DT and XGBoost models emerge as the top performers, achieving the lowest total RSs of 6 and 5, respectively, reflecting their high predictive accuracy across all metrics. The GBR and RF models also perform well, with total RS of 10 and 14, respectively, indicating strong performance but slightly lower performance than that of DT and XGBoost. With a total RSs of 18, the GPR model still shows good accuracy but outperforms the aforementioned models. The symbolic regression and SVM models, with total RSs of 23 and 30, respectively, have significantly lower accuracies, with the SVM model performing the worst in this category.

A similar trend is observed for STS in Fig. 11 (b). DT and XGBoost achieve the highest accuracy again, with the lowest total RSs of 7 and 9, respectively. The GBR and RF closely follow with total RSs of 15 and 18, showing strong but slightly lower performance than DT and XGBoost. GPR, with a total RS of 21, performs moderately well but is outperformed by the other models. The SVM and symbolic regression models again yield the lowest accuracy, with total RSs of 28 and 33, respectively, with symbolic regression being the least effective in predicting STS.

In summary, the DT and XGBoost models consistently rank as the most accurate for both the CS and the STS, whereas the SVM and symbolic regression ranks are the least accurate. This evaluation highlights the effectiveness of DT and XGBoost in modeling complex relationships within the data, making them the preferred choices for predicting concrete properties.

#### 3.2.3 Comparison of the measured and developed ML algorithms

**Fig. 12:**
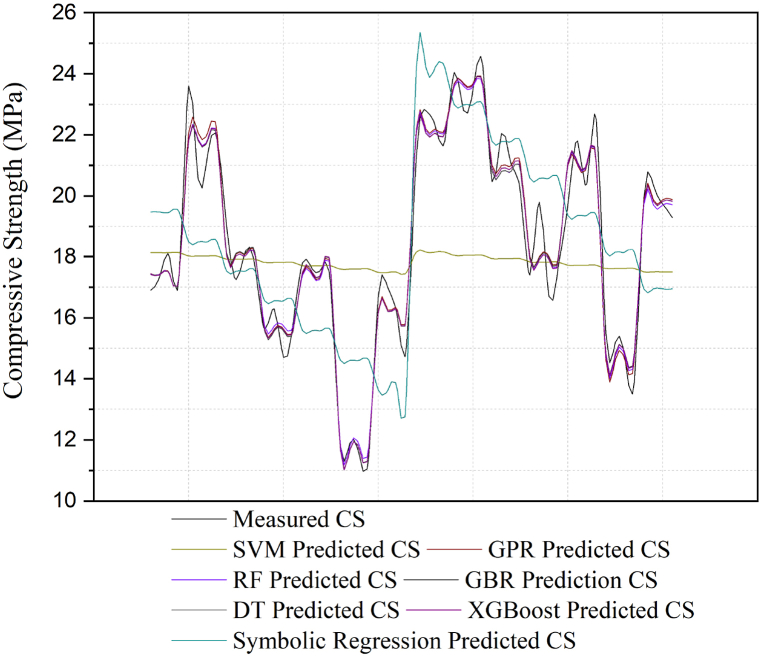
Comparison of the measured CS in the test with the predictions from the models.

**Fig. 13:**
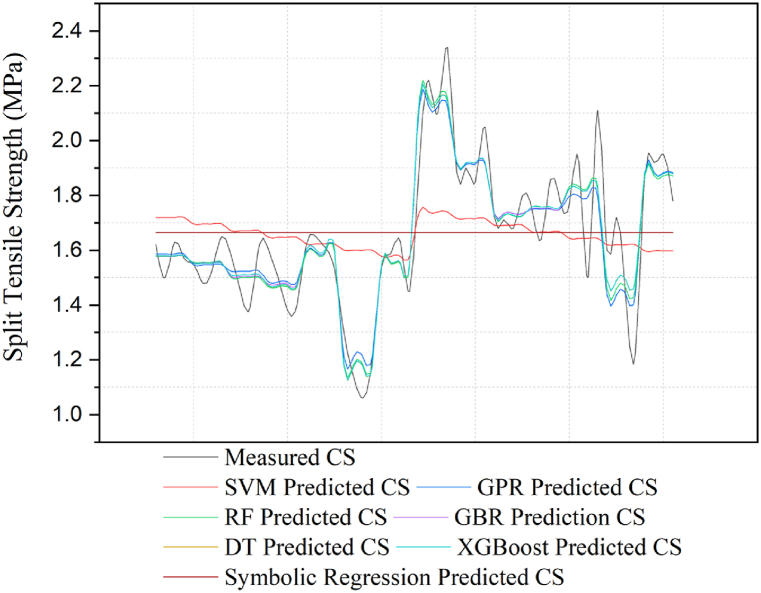
Comparison of the measured STSs in the tests with the predictions from the models.

The following Fig. 12, Fig. 13-line graphs compare concrete's predicted CS and STS values using various machine learning models against the measured compressive strength. The graph includes predictions from the SVM, GPR, RF, GBR, DT, XGBoost, and symbolic regression models, each representing a distinct colored line. The black line represents the measured CS values, which serve as the measured for comparison.

Fig. 12 illustrates that SVM (yellow line) and symbolic regression (light green line) show significant deviations from the measured values, indicating poor predictive performance. In contrast, DT (gray line), XGBoost (cyan line), RF (purple line), and GBR (light blue line) closely follow the measured CS values, demonstrating high predictive accuracy. GPR (brown line) performs moderately, with minor deviations. Overall, DT and XGBoost prove the most effective in predicting concrete compressive strength, while SVM and symbolic regression perform the weakest.

Fig. 13 compares the measured and predicted split tensile strength (STS) values using various machine learning models. The SVM (red line) and symbolic regression (light green line) show poor performance, with significant deviations from the measured values, particularly underestimating strength. In contrast, DT (gray line) and XGBoost (cyan line) closely follow the measured STS, indicating high accuracy. RF (green line) and GBR (purple line) also perform well, with minor deviations, while GPR (light blue line) shows moderate accuracy. Overall, DT and XGBoost provide the most accurate predictions, while SVM and symbolic regression are the least accurate.

**Fig. 14:**
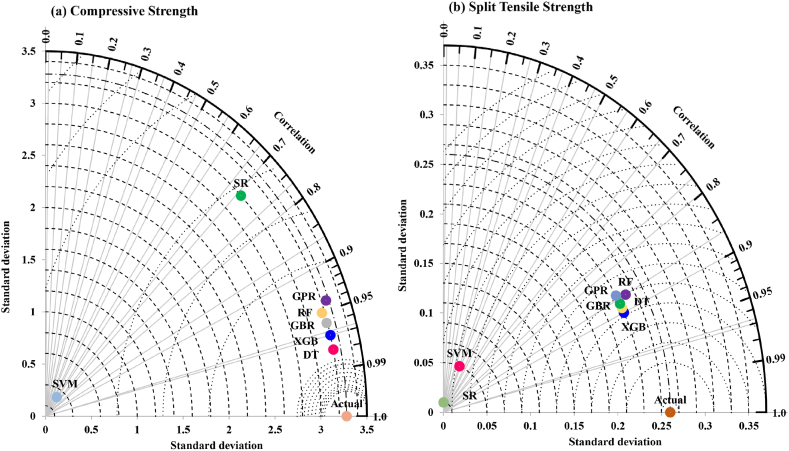
Taylor diagrams for predicting, (a) compressive strength and (b) split tensile strength.

In addition, the Taylor diagrams shown in Fig. 14(a and b) compare the performance of different machine learning algorithms for predicting two mechanical properties: compressive strength and split tensile strength**.** It enables a clear comparison of multiple models by simultaneously plotting all metrics, with models closer to the actual point representing higher accuracy [79].

In the compressive strength shown in Fig. 14 (a), the algorithms GPR, RF, GBR, XGB, and DT again exhibit high correlation and lower standard deviation, indicating strong performance in predicting compressive strength. SR and SVM show lower correlation and higher standard deviation, implying less accurate predictions for this mechanical property. Overall, GPR and RF appear to be the best-performing models for both properties, while SR and SVM are less effective. Similarly, In the split tensile strength diagram shown in Fig. 14 (b), the algorithms GPR, RF, GBR, XGB, and DT are grouped with high correlation coefficients (>0.95) and relatively low standard deviations, indicating strong predictive performance. In contrast, SVM is positioned farther from the actual point, suggesting lower accuracy in its predictions.

### 3.3 Cost analysis of WIS concrete mixtures

The cost analysis is a financial evaluation tool used to assess the total costs associated with a project, product, or service, often comparing these costs against potential benefits or revenues. In the context of concrete mixtures incorporating WIS, cost analysis involves evaluating how including WIS as a substitute for traditional materials impacts the overall cost of producing concrete. Table 4 illustrates the total cost in Bangladeshi Taka (BDT) for different concrete mixtures, each with varying amounts of WIS included as a replacement in the mixtures. The production and transportation unit costs for concrete materials were obtained from previous research using the latest data and aligned with Bangladesh standards [80]. The production cost of WIS is zero, with only transportation costs incurred since it was sourced locally. To calculate the percentage decrease in total cost as the WIS amount increases, the following Equation (11) is used,

(11) $$PI=\frac{\mathrm{Cos}\;t_{WIS0}}{\mathrm{Cos}\;t_{WIS0}-\mathrm{Cos}\;t_{WISX}}$$

where PI is percentage increase, WIS0 cost is the total cost when no WIS is used (8465.8094 BDT), and WISX cost is the total cost for each corresponding WIS amount.

**Table 4:** WIS concrete mix cost analysis.

**Unit Cost (BDT/kg)**			**Concrete MIX ID**	**Mixture cost (BDT/m**^**3**^**)**					**Total Cost (BDT)**
**Material**	**Costing Phase**			**Cement**	**FA**	**CA**	**WSI**	**Water**	
	**Production**	**Transportation**							
Cement	9	0.34	WIS0	2445.2	1101.0	4909.1	0	10.36	8465.8094
CA	5	0.26	WIS5	2445.2	1045.8	4909.1	3.45	10.36	8414.0504
FA	2.17	0.22	WIS10	2445.2	990.89	4909.1	6.915	10.36	8362.5454
WIS	0	0.15	WIS15	2445.2	935.92	4909.1	10.365	10.36	8311.0254
Water	0.088		WIS20	2445.2	880.71	4909.1	13.815	10.36	8259.2664
			WIS25	2445.2	825.74	4909.1	17.28	10.36	8207.7614
			WIS30	2445.2	770.77	4909.1	20.73	10.36	8156.2414

As the WIS content in the mixture increases, the total cost progressively decreases. For example, increasing the WIS content from 0 % to 30 % results in a total cost reduction of approximately 3.65 %. This decline in cost is attributed to the lower expense associated with WIS than with conventional fine aggregates. The percentage decrease in total cost for each step increase in WIS content is as follows: 0.61 % for WIS5, 1.22 % for WIS10, 1.83 % for WIS15, 2.44 % for WIS20, 3.05 % for WIS25, and 3.65 % for WIS30. This analysis highlights the economic advantage of using WIS in concrete production, demonstrating that higher WIS content can lead to significant cost savings while contributing to more sustainable construction practices.

Additionally, the cost index is a critical component in this study as it offers a precise metric for evaluating the cost-effectiveness of various concrete mixes by measuring the expense required to achieve a specific compressive strength. By calculating the cost per MPa of strength, researchers can determine which concrete mixtures provide the most efficient balance between cost and performance, making it a key factor in selecting sustainable and economical construction materials. This metric, referred to as the cost index of the concrete mix, is determined by the costs incurred to reach 1 MPa of compressive strength at 28 days. Understanding and applying the cost index is vital for making informed decisions that align with budgetary constraints and the desired structural performance, ultimately contributing to more sustainable construction practices. The calculation of this index is fundamental to ensuring that the chosen mix designs meet the technical requirements and adhere to economic efficiency, thereby optimizing resource allocation in construction projects. The cost index of the concrete mixture was obtained via Equation (12) [81].

(12) $$CI=\frac{Cost}{C}$$

where CI = the cost index, cost = the cost of producing a 1 m^3^ concrete mixture (BDT), and Cs = the compressive strength attained by the concrete mix after 28 days (MPa).

**Fig. 15:**
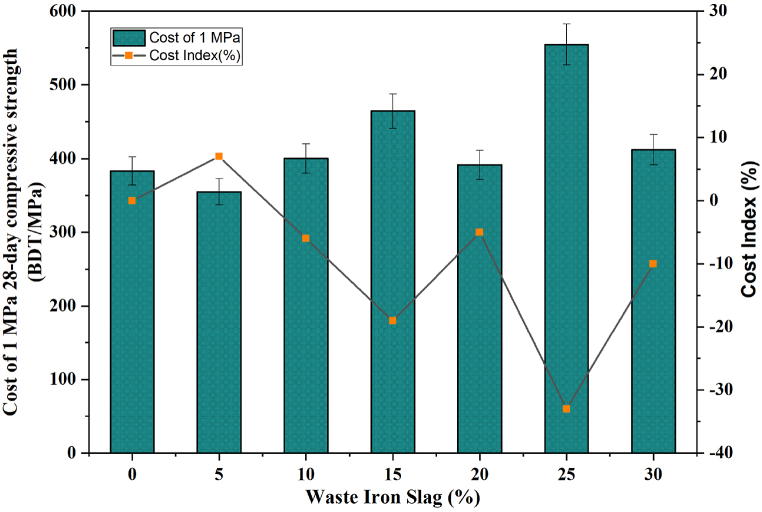
Cost index of the WIS concrete mixtures.

The analysis of the cost of producing 1 MPa of 28-day compressive strength with varying percentages of WIS reveals significant insights into cost efficiency, as presented in Fig. 15. Initially, at 0 % WIS, the cost is 383.9 BDT/MPa, which serves as the control mix. When 5 % WIS is introduced, the cost slightly decreases to 355.2 BDT/MPa, but the cost index increases by approximately 7 %, indicating a marginal increase in overall costs despite the reduced cost per MPa. This trend continues with 10 % WIS, where the cost per MPa increases to 400.12 BDT/MPa, and the cost index increases further to approximately 10 %. The cost continues to increase at 15 % and 20 % WIS, reaching 464.3 BDT/MPa and 391.4 BDT/MPa, respectively, with a corresponding cost index increase of approximately 15 %. However, at 25 % WIS, there is a significant decrease in cost to 554.5 BDT/MPa, and the cost index decreases to approximately −33 %, reflecting substantial cost savings. Finally, at 30 % WIS, the cost increases sharply to 411.9 BDT/MPa, and the cost index increases but remains below the measured at approximately −10 %, indicating that while costs rise compared with those at 25 % WIS, they are still lower than those without WIS. Despite the initial increase in costs at lower WIS percentages, 25 % WIS results in substantial cost savings, as evidenced by the dramatic reduction in the cost index to −33 %, making it the most efficient in terms of reducing overall costs for producing compressive strength. This analysis highlights the importance of finding the right balance in the percentage of WIS used to maximize cost savings while maintaining the desired compressive strength.

## 4 Limitations and future work

The study was limited by a relatively small dataset, which may restrict the generalizability of the findings across different conditions and WIS sources. Additionally, the experimental data were confined to specific mix designs and curing conditions, potentially affecting the applicability of the results to other scenarios. Furthermore, the study focused primarily on compressive and tensile strength, neglecting other critical mechanical properties such as flexural strength and creep behavior, as well as long-term durability aspects like resistance to chemical attacks, freeze-thaw cycles, and carbonation. These limitations could affect the broader adoption of WIS as a sustainable material in concrete production.

To address these limitations, future research should focus on expanding the dataset to include a wider array of mix designs, WIS sources, and environmental conditions. Furthermore, Advanced ML techniques, including deep learning and hybrid modeling approaches, should also be explored to improve predictive accuracy and uncover intricate interactions between concrete components and their effects on strength and durability. Investigating these factors will pave the way for a more holistic understanding of WIS as a viable, sustainable material in the construction industry.

## 5 Concluding remarks

In the pursuit of sustainable construction practices, the integration of waste materials, such as WIS, into concrete production presents a promising strategy for minimizing environmental impact and conserving natural resources. This study provides valuable insights into the potential of WIS as a supplementary material in the construction industry through experimental evaluation and the application of machine learning models to predict the mechanical properties of WIS-incorporated concrete. By systematically analyzing compressive strength and other related properties, the research contributes to the growing body of knowledge on sustainable construction materials and underscores the importance of optimizing material utilization through advanced predictive techniques. The conclusions derived from this study are as follows:

• The study revealed that the optimal replacement level of WIS, while maintaining adequate strength properties, ranged between 10 % and 20 %. Beyond this range, a noticeable decrease in strength was observed, suggesting that the increased incorporation of WIS could adversely affect the structural integrity of the concrete.
• The inclusion of WIS in concrete mixtures presents a viable solution for waste management in the iron and steel industry, contributing to more sustainable construction practices by reducing the reliance on natural aggregates.
• DT and XGBoost models exhibited the highest predictive accuracy, achieving an R^2^ value of 0.95135 for both compressive and split tensile strengths. These models effectively modeled the complex, non-linear relationships within the dataset.
• The application of machine learning provides a robust framework for predicting concrete strength properties, allowing for the optimization of mix designs with WIS, which is particularly valuable for developing sustainable construction materials.
• Integrating a ranking score index and cost analysis further validated the machine learning predictions, ensuring that the incorporation of WIS is both technically and economically viable.
• The experimental results align well with the predictions made by the machine learning models, demonstrating the models' reliability in practical applications. The strong correlation between the experimental and predicted values indicates that machine learning can effectively complement experimental research, reducing the need for extensive physical testing.
• The findings of this study suggest that machine learning algorithms, especially DT and XGBoost, can accurately capture the non-linear relationships between the input variables (such as WIS content, water-cement ratio, and aggregate properties) and the resulting concrete strength. This capability is crucial for optimizing mix designs more efficiently and cost-effectively.
• Taylor diagram analysis revealed that GPR and RF models also demonstrated robust predictive performance, particularly for compressive and tensile strengths.
• WIS incorporation reduces concrete production costs by up to 3.65 % at 30 % replacement, demonstrating economic and environmental benefits.
• Expanded datasets and advanced machine learning techniques are needed to predict long-term durability better and explore other mechanical properties like flexural strength.

## CRediT authorship contribution statement

**Matiur Rahman Raju:** Writing – original draft, Formal analysis, Data curation, Conceptualization. **Syed Ishtiaq Ahmad:** Writing – review & editing, Supervision, Resources, Project administration, Investigation, Funding acquisition. **Md Mehedi Hasan:** Writing – review & editing, Visualization, Validation, Software, Data curation. **Noor Md. Sadiqul Hasan:** Writing – review & editing, Supervision, Investigation, Formal analysis. **Md Monirul Islam:** Writing – review & editing, Supervision, Resources, Project administration, Funding acquisition. **Md. Abdul Basit:** Writing – review & editing, Validation, Data curation. **Ishraq Tasnim Hossain:** Writing – review & editing, Visualization, Validation, Data curation. **Saif Ahmed Santo:** Writing – review & editing, Validation, Data curation. **Md Shahrior Alam:** Writing – review & editing, Visualization, Validation, Software, Data curation. **Mahfuzur Rahman:** Writing – review & editing, Supervision, Resources, Funding acquisition, Formal analysis,Validation, Software.

## Data availability statement

The actual and predicted strength data of this article are included in Appendix A. All data, models, or codes generated or used during the study are available from the corresponding author by request. Dataset link: https://github.com/Sirfowahid/Iron_Slug.

## Funding

This research received no external funding.

## Declaration of competing interest

The authors declare that they have no known competing financial interests or personal relationships that could have appeared to influence the work reported in this paper.

## Figures and Tables

**Table A1:** Measured and predicted values of compressive strength.

**Measured (MPa)**	**Predicted (MPa)**						
	**SVM**	**GPR**	**RF**	**GBR**	**DT**	**XGBoost**	**Symbolic Regression**
16.900	18.132	17.438	17.433	17.427	17.418	17.418	19.476
17.400	18.132	17.438	17.433	17.427	17.418	17.418	19.476
17.970	18.132	17.438	17.433	17.427	17.418	17.418	19.476
17.400	18.132	17.438	17.433	17.427	17.418	17.418	19.476
23.600	18.026	22.063	21.856	21.804	21.838	21.837	18.500
20.550	18.026	22.063	21.856	21.804	21.838	21.837	18.500
21.350	18.026	22.063	21.856	21.804	21.838	21.837	18.500
21.850	18.026	22.063	21.856	21.804	21.838	21.837	18.500
19.010	17.918	18.094	18.031	18.116	18.030	18.030	17.527
17.250	17.918	18.094	18.031	18.116	18.030	18.030	17.527
18.200	17.918	18.094	18.031	18.116	18.030	18.030	17.527
17.660	17.918	18.094	18.031	18.116	18.030	18.030	17.527
15.600	17.809	15.625	15.726	15.563	15.600	15.600	16.555
16.300	17.809	15.625	15.726	15.563	15.600	15.600	16.555
14.700	17.809	15.625	15.726	15.563	15.600	15.600	16.555
15.800	17.809	15.625	15.726	15.563	15.600	15.600	16.555
17.800	17.700	17.438	17.396	17.485	17.500	17.500	15.578
17.600	17.700	17.438	17.396	17.485	17.500	17.500	15.578
17.600	17.700	17.438	17.396	17.485	17.500	17.500	15.578
17.000	17.700	17.438	17.396	17.485	17.500	17.500	15.578
11.800	17.592	11.688	11.808	11.689	11.675	11.676	14.606
11.900	17.592	11.688	11.808	11.689	11.675	11.676	14.606
11.500	17.592	11.688	11.808	11.689	11.675	11.676	14.606
11.500	17.592	11.688	11.808	11.689	11.675	11.676	14.606
16.740	17.487	16.250	16.213	16.199	16.208	16.208	13.634
16.980	17.487	16.250	16.213	16.199	16.208	16.208	13.634
16.030	17.487	16.250	16.213	16.199	16.208	16.208	13.634
15.080	17.487	16.250	16.213	16.199	16.208	16.208	13.634
21.300	18.150	22.188	22.137	22.056	22.050	22.050	24.204
22.800	18.150	22.188	22.137	22.056	22.050	22.050	24.204
22.300	18.150	22.188	22.137	22.056	22.050	22.050	24.204
21.800	18.150	22.188	22.137	22.056	22.050	22.050	24.204
24.040	18.044	23.688	23.592	23.660	23.655	23.654	22.990
22.800	18.044	23.688	23.592	23.660	23.655	23.654	22.990
23.510	18.044	23.688	23.592	23.660	23.655	23.654	22.990
24.270	18.044	23.688	23.592	23.660	23.655	23.654	22.990
20.470	17.936	21.000	20.813	20.811	20.905	20.905	21.782
22.040	17.936	21.000	20.813	20.811	20.905	20.905	21.782
21.030	17.936	21.000	20.813	20.811	20.905	20.905	21.782
20.080	17.936	21.000	20.813	20.811	20.905	20.905	21.782
17.400	17.827	18.000	17.917	17.971	17.900	17.900	20.574
19.800	17.827	18.000	17.917	17.971	17.900	17.900	20.574
16.700	17.827	18.000	17.917	17.971	17.900	17.900	20.574
17.700	17.827	18.000	17.917	17.971	17.900	17.900	20.574
19.700	17.719	21.000	21.051	21.085	21.100	21.100	19.360
21.800	17.719	21.000	21.051	21.085	21.100	21.100	19.360
20.400	17.719	21.000	21.051	21.085	21.100	21.100	19.360
22.500	17.719	21.000	21.051	21.085	21.100	21.100	19.360
15.500	17.611	14.625	14.743	14.841	14.825	14.825	18.152
15.200	17.611	14.625	14.743	14.841	14.825	14.825	18.152
14.700	17.611	14.625	14.743	14.841	14.825	14.825	18.152
13.900	17.611	14.625	14.743	14.841	14.825	14.825	18.152
19.930	17.505	19.875	19.713	19.824	19.830	19.830	16.943
20.360	17.505	19.875	19.713	19.824	19.830	19.830	16.943
19.730	17.505	19.875	19.713	19.824	19.830	19.830	16.943
19.300	17.505	19.875	19.713	19.824	19.830	19.830	16.943

**Table A2:** Measured and predicted values of split tensile strength.

**Measured (MPa)**	**Predicted (MPa)**						
	**SVM**	**GPR**	**RF**	**GBR**	**DT**	**XGBoost**	**Symbolic Regression**
1.620	1.720	1.586	1.578	1.580	1.580	1.580	1.665
1.500	1.720	1.586	1.578	1.580	1.580	1.580	1.665
1.630	1.720	1.586	1.578	1.580	1.580	1.580	1.665
1.570	1.720	1.586	1.578	1.580	1.580	1.580	1.665
1.550	1.696	1.547	1.556	1.553	1.553	1.553	1.665
1.480	1.696	1.547	1.556	1.553	1.553	1.553	1.665
1.530	1.696	1.547	1.556	1.553	1.553	1.553	1.665
1.650	1.696	1.547	1.556	1.553	1.553	1.553	1.665
1.580	1.671	1.523	1.500	1.504	1.510	1.510	1.665
1.450	1.671	1.523	1.500	1.504	1.510	1.510	1.665
1.390	1.671	1.523	1.500	1.504	1.510	1.510	1.665
1.620	1.671	1.523	1.500	1.504	1.510	1.510	1.665
1.600	1.647	1.484	1.466	1.476	1.470	1.470	1.665
1.500	1.647	1.484	1.466	1.476	1.470	1.470	1.665
1.380	1.647	1.484	1.466	1.476	1.470	1.470	1.665
1.400	1.647	1.484	1.466	1.476	1.470	1.470	1.665
1.620	1.623	1.594	1.590	1.603	1.603	1.602	1.665
1.650	1.623	1.594	1.590	1.603	1.603	1.602	1.665
1.610	1.623	1.594	1.590	1.603	1.603	1.602	1.665
1.530	1.623	1.594	1.590	1.603	1.603	1.602	1.665
1.300	1.600	1.211	1.182	1.175	1.175	1.176	1.665
1.140	1.600	1.211	1.182	1.175	1.175	1.176	1.665
1.060	1.600	1.211	1.182	1.175	1.175	1.176	1.665
1.200	1.600	1.211	1.182	1.175	1.175	1.176	1.665
1.540	1.578	1.551	1.549	1.552	1.553	1.553	1.665
1.590	1.578	1.551	1.549	1.552	1.553	1.553	1.665
1.630	1.578	1.551	1.549	1.552	1.553	1.553	1.665
1.450	1.578	1.551	1.549	1.552	1.553	1.553	1.665
1.910	1.740	2.125	2.154	2.142	2.143	2.142	1.665
2.220	1.740	2.125	2.154	2.142	2.143	2.142	1.665
2.100	1.740	2.125	2.154	2.142	2.143	2.142	1.665
2.340	1.740	2.125	2.154	2.142	2.143	2.142	1.665
1.880	1.716	1.914	1.919	1.919	1.920	1.920	1.665
1.900	1.716	1.914	1.919	1.919	1.920	1.920	1.665
1.850	1.716	1.914	1.919	1.919	1.920	1.920	1.665
2.050	1.716	1.914	1.919	1.919	1.920	1.920	1.665
1.720	1.691	1.734	1.726	1.734	1.728	1.728	1.665
1.710	1.691	1.734	1.726	1.734	1.728	1.728	1.665
1.680	1.691	1.734	1.726	1.734	1.728	1.728	1.665
1.800	1.691	1.734	1.726	1.734	1.728	1.728	1.665
1.750	1.667	1.750	1.758	1.752	1.758	1.757	1.665
1.640	1.667	1.750	1.758	1.752	1.758	1.757	1.665
1.860	1.667	1.750	1.758	1.752	1.758	1.757	1.665
1.780	1.667	1.750	1.758	1.752	1.758	1.757	1.665
1.770	1.643	1.797	1.832	1.824	1.825	1.825	1.665
1.920	1.643	1.797	1.832	1.824	1.825	1.825	1.665
1.500	1.643	1.797	1.832	1.824	1.825	1.825	1.665
2.110	1.643	1.797	1.832	1.824	1.825	1.825	1.665
1.600	1.620	1.438	1.460	1.491	1.490	1.490	1.665
1.720	1.620	1.438	1.460	1.491	1.490	1.490	1.665
1.420	1.620	1.438	1.460	1.491	1.490	1.490	1.665
1.220	1.620	1.438	1.460	1.491	1.490	1.490	1.665
1.870	1.598	1.883	1.871	1.880	1.880	1.880	1.665
1.920	1.598	1.883	1.871	1.880	1.880	1.880	1.665
1.950	1.598	1.883	1.871	1.880	1.880	1.880	1.665
1.780	1.598	1.883	1.871	1.880	1.880	1.880	1.665
